# Diagnostic value of TWEAK for predicting active lupus nephritis in patients with systemic lupus erythematosus: a systematic review and meta-analysis

**DOI:** 10.1080/0886022X.2020.1853568

**Published:** 2020-12-13

**Authors:** Hao-Yang Ma, Shuang Chen, Wei-Dong Cao, Cui-Ting Min

**Affiliations:** a Department of Pediatrics, Medical School of Southeast University, Nanjing, China; b Jiangsu Key Laboratory of Pediatrics, Nanjing Medical University, Nanjing, China; c Nanjing Key Laboratory of Pediatrics, Children’s Hospital of Nanjing Medical University, Nanjing, China

**Keywords:** TWEAK, lupus nephritis, diagnosis, meta-analysis

## Abstract

**Purpose:**

Accumulative studies showed that tumor necrosis factor (TNF)-like weak inducer of apoptosis (TWEAK) was up-regulated in the blood and urine from patients diagnosed with lupus nephritis (LN) and that it might be used as a novel biomarker for active LN. This meta-analysis aimed to determine the diagnostic value of TWEAK in active LN.

**Methods:**

We searched the Cochrane Library, Embase, PubMed, Springer, Wanfang and CNKI databases for articles published up to 20 August 2020. The diagnostic capacity of TWEAK for active LN was assessed using pooled sensitivity and specificity, positive and negative likelihood ratios (PLR and NLR), diagnostic odds ratio (DOR), and area under the receiver operating characteristic curve (AUC). Quality assessment and publication bias were also evaluated. STATA 11.0 and Meta-Disc 1.4 were used to perform these analyses.

**Results:**

Nine cross-sectional studies were included in this meta-analysis. The overall pooled sensitivity of TWEAK for the diagnosis of active LN was 0.69 (95% CI, 0.63–0.75), and specificity was 0.77 (95% CI, 0.71–0.82). The overall pooled PLR and NLR were 3.31 (95% CI, 2.05–5.35) and 0.38 (95% CI, 0.26–0.55), respectively, with a DOR of 10.89 (95% CI, 6.73–17.63) and AUC (SE) of 0.8276 (0.0289). Deeks’ funnel plot revealed that the publication bias was insignificant in the study (*p* = .32).

**Conclusions:**

Our results suggest that TWEAK might be a potential biomarker for patients with active LN. Future cross-sectional and longitudinal studies are needed to confirm its diagnostic value, as well as to establish more definite cutoff for active LN.

## Introduction

As a common clinical manifestation of systemic lupus erythematosus (SLE), lupus nephritis (LN) is characterized by immune complex deposition, renal microvascular lesions, inflammation, proteinuria, hematuria and progressive renal dysfunction [1,2]. It affects approximately 60% of patients and is a major risk factor for morbidity and mortality in SLE [3,4]. Approximately 22% of patients with LN might develop end-stage renal disease (ESRD) within 15 years and this risk is greatest in the first five years, suggesting that early identification and intervention are critical in the preservation of renal function [5]. Percutaneous renal biopsy is the gold standard for the diagnosis of LN and provides guidance for risk stratification and treatment [6]. Routine monitoring for the progression of LN is done with complement levels, anti-dsDNA, serial creatinine, urinary protein/creatinine ratio, and urinalysis [7,8]. However, the current biomarkers for the assessment of LN activity lack sensitivity and specificity. These indicators cannot always ideally correlate with renal activity and damage [9]. Moreover, renal biopsy is an invasive procedure which is impractical to utilize on a serial basis to monitor LN flares [10]. Thus, finding reliable and specific biomarkers is paramount for clinicians to monitor disease activity and/or renal involvement in patients with SLE.

Tumor necrosis factor (TNF)-like weak inducer of apoptosis (TWEAK), a member of the TNF superfamily ligands, is a multi-functional cytokine which binds to its receptor known as fibroblast growth factor-inducible 14 kDa protein (FN14) [11]. TWEAK is expressed in innate immune cells such as natural killer cells, macrophages and dendritic cells, and is thought to play a critical role in immune modulation [12]. Preclinical studies suggested that the activation of TWEAK/FN14 signaling pathway was involved in the pathogenesis of LN and that the inhibition of TWEAK could improve glomerulonephritis in murine models of lupus [13–15]. Compared with healthy controls, patients with LN had higher glomerular and tubulointerstitial expressions of TWEAK [16]. The expression of TWEAK was also elevated in peripheral blood mononuclear cells from patients with LN and was positively correlated with disease activity [17].

Emerging evidence showed that TWEAK was up-regulated in the blood and urine from patients diagnosed with LN and that it might be used as a novel biomarker for active LN [18,19]. However, there existed a wide range of variability in TWEAK’s diagnostic performance for LN [20,21]. The present meta-analysis aims to accumulate current literature knowledge in the field to determine the diagnostic accuracy of TWEAK in the prediction of active LN in patients with SLE.

## Materials and methods

### Search strategy

A comprehensive literature search was performed in the Cochrane Library, Embase, PubMed, Springer, Chinese National Knowledge Infrastructure (CNKI) and Wanfang databases up to 20 August 2020, using one or a combination of the following terms: TNF-like weak inducer of apoptosis, TWEAK, systemic lupus erythematosus, SLE, lupus nephritis. There was no language limitation in the literature searching. In addition, the relevant references and cited papers were searched manually to identify additional studies meeting the inclusion criteria.

### Inclusion and exclusion criteria

The study inclusion criteria were as follows: (1) the study participants were human; (2) patients diagnosed with SLE according to the 1982 and 1997 American College of Rheumatology criteria (ACR-1982, 1997) and the 2012 Systemic Lupus International Collaborating Clinics (SLICC-2012) [22–24]; (3) SLE patients with or without active LN determined by renal SLE disease activity index (rSLEDAI) scoring or kidney biopsy; (4) availability of indexes including sensitivity, specificity, diagnostic thresholds for TWEAK, or data from which true positive (TP), false positive (FP), false negative (FN) and true negative (TN) could be obtained or calculated.

The study exclusion criteria were as follows: (1) studies not focusing on the diagnostic performance of TWEAK in predicting active LN in patients with SLE; (2) case reports and reviews; (3) studies without mandatory predictive variables including the area under the receiver operating characteristic curve (AUC), sensitivity and specificity.

### Data extraction and quality assessment

All data were extracted independently by two authors using a paper data extraction form. The accuracy of the extracted data was further confirmed by a third author. The extracted information included: (1) characteristics of the included studies: author and year of publication, country, study design, participants, baseline serum creatinine (Scr) level, definition of active LN and key findings; (2) diagnostic accuracies of the included studies for TWEAK to predict active LN: sample type (urine or serum), detection method, TWEAK cutoff value, sensitivity, specificity and AUC in each study. If a study lacked basic data, we calculated the TP/FP/FN/TN according to the following formulas: sensitivity = TP/(TP + FN), specificity = TN/(FP + TN), (SLE with active LN) + (SLE without active LN) = TP + FP + TN + FN and filled in the 2 × 2 table. The risk of bias was assessed using guidelines proposed in Quality Assessment of Diagnostic Accuracy Studies 2 (QUADAS-2) [25].

### Statistical analysis

We performed the analysis using Stata 11.0 software (Stata Corporation, College Station, TX) and Meta-Disc version 1.4 (Universidad Complutense, Madrid, Spain). Heterogeneity among the studies was assessed with the Cochran Q test and I-squared (I^2^) statistics test. If the heterogeneity was statistically significant (*p* < .05 or I^2^ > 50%), the random-effects model was used to calculate the pooled effect sizes such as sensitivity, specificity, positive likelihood ratio (PLR), negative likelihood ratio (NLR), and diagnostic odds ratio (DOR); otherwise, the fixed-effects model was employed. The AUC and Cochrane indices (Q*) were calculated. An AUC ≥ 0.70 defines a useful risk predictor. We also tested the publication bias using Deeks’ funnel plot method.

## Results

### Study selection

**Figure 1.**
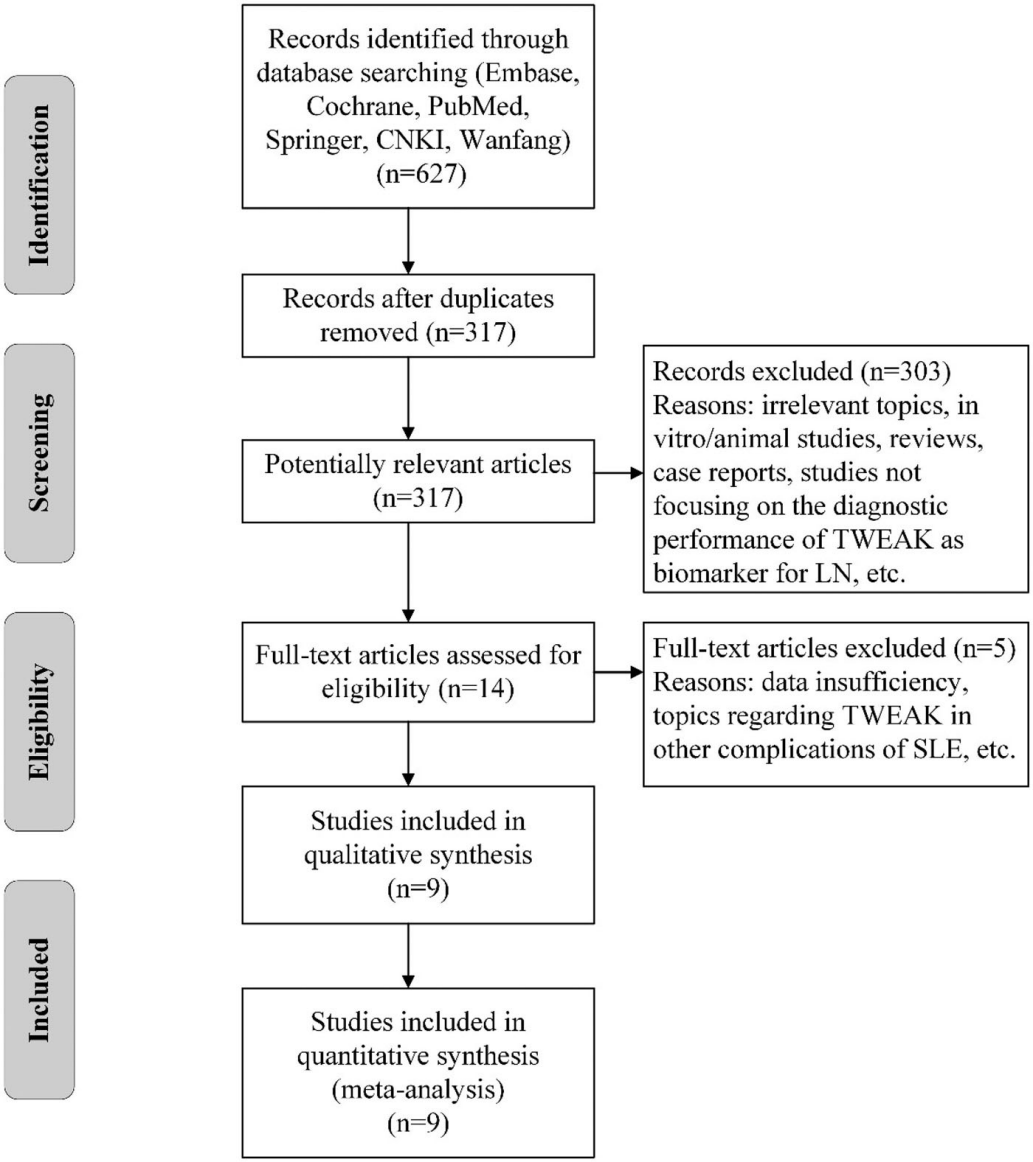
Flowchart of paper selection. TWEAK: tumor necrosis factor (TNF)-like weak inducer of apoptosis; LN: lupus nephritis; SLE: systemic lupus erythematosus.

A flowchart of the selection process is shown in Figure 1. After discarding the duplicate studies and initial screening, 14 studies were selected for full-text examination. Two studies were excluded due to data insufficiency [17,26]. Three studies were excluded because of not focusing on the diagnostic value of TWEAK in LN [27–29]. Nine studies fulfilled the inclusion criteria and were ultimately included in this analysis [20,21,30–36].

### Characteristics of the included studies

Characteristics of the included studies are summarized in Table 1. The enrolled studies were conducted in different continents, varying from North America, Asia, Africa and Europe, with the publication years ranging from 2009 to 2020. Most of the studies were of cross-sectional design. As expected, a majority of the participants with SLE were female patients. The presence of active LN was determined by rSLEDAI score of at least > 0 or renal biopsy. Another study conducted by XW Dong et al. in 2018 investigated the level of TWEAK in proteinuria detection in patients with LN. Since proteinuria is a component of rSLEDAI scoring system, the study was also included. All the eligible studies focused on the diagnostic performance of TWEAK as biomarker for active LN.

**Table 1.** Characteristics of the included studies.

Author, year	Country	Study design	Participants, female (%)	Baseline Scr (mg/dl)	Definition of active LN	Key findings
Noa Schwartz, et al. 2009	USA	Multicenter cohort study. Cross-sectional and longitudinal studies.	SLE + LN: *n* = 30. F: 93%. SLE-LN: *n* = 49. F: 88%.	SLE + LN: 0.85 (0.60–1.20)^b^. SLE–LN: 0.70 (0.60–0.80)^b^.	rSLEDAI scor*e* > 0.	High uTWEAK indicated the presence of LN and reflected renal disease activity in the follow-up. TWEAK signaling pathway might be involved in the pathogenesis of LN.
Jiazhen Tan, et al. 2009	China	Cross-sectional study.	SLE + LN: *n* = 34. F: 97.1%. SLE-LN: *n* = 12. F: 75%.	SLE + LN: 1.28 ± 0.11. SLE–LN: 1.04 ± 0.22.	rSLEDAI scor*e* > 4.	The level of uTWEAK reflected renal disease activity and could be a novel biomarker of LN.
S. Marzouk, et al. 2011	Egypt	Cross-sectional study.	SLE + LN: *n* = 50. SLE-LN: *n* = 23. F (total): 96%.	SLE + LN: 0.80 ± 0.30. SLE–LN: 0.56 ± 0.20.	rSLEDAI scor*e* > 4.	uTWEAK along with other urinary biomarkers (OPG, MCP-1, and IL-8) correlated with renal involvement in patients with SLE.
Jung-Yoon Choe, et al. 2016	Republic of Korea	Cross-sectional study.	SLE + LN: *n* = 32. F: 100%. SLE-LN: *n* = 38. F: 100%.	Not reported separately. SLE + LN and SLE–LN: 0.8 ± 0.8.	rSLEDAI scor*e* > 0.	sTWEAK might be a biomarker candidate that reflected renal involvement in patients with SLE.
Fabiola Reyes Martínez, et al. 2017	Mexico	Cross-sectional study.	SLE + LN: *n* = 11. F: 81.8%. SLE-LN: *n* = 11. F: 90.9%.	SLE + LN: 1.60 ± 1.53. SLE–LN: 0.72 ± 0.16.	Renal activity was proven by kidney biopsy.	uTWEAK could distinguish renal activity with higher sensitivity and specificity compared with the commonly used biomarkers.
XW Dong, et al. 2018	China	Cross-sectional study.	SLE + LN: *n* = 48. F: 85.4%. SLE-LN: *n* = 22. F: 90.9%.	SLE + LN: 1.04 ± 0.48. SLE–LN: 0.86 ± 0.22.	rSLEDAI scor*e* > 0.	uTWEAK combined with uMCP-1 could discriminate severe LN patients and predict LN renal prognosis.
XW Dong, et al. 2018^a^	China	Cross-sectional study.	SLE + LN: *n* = 39. F: 89.7%. SLE-LN: *n* = 20. F: 90%.	NR.	24-hour urine proteinuri*a* > 300 mg/day, further confirmed by kidney biopsy.	uTWEAK was elevated in patients with active LN and correlated with 24-hour urine proteinuria.
M. N. Salem, et al. 2018	Egypt	Cross-sectional study.	SLE + LN: *n* = 14. F: 96.7%. SLE-LN: *n* = 30. F: 78.6%.	SLE + LN: 2.10 ± 1.90. SLE–LN: 0.84 ± 0.68.	rSLEDAI scor*e* > 4.	uTWEAK was a sensitive biomarker for early detection of active LN.
S Mirioglu, et al. 2020	Turkey	Cross-sectional study.	SLE + LN: *n* = 15. F: 73.3%. SLE-LN: *n* = 15. F: 93.3%.	SLE + LN: 0.7 (0.6–1.63)^b^. SLE–LN: NR.	rSLEDAI scor*e* > 0.	sTWEAK was helpful in distinguishing patients with active LN. uTWEAK was not able to discriminate active LN.

^a^This study compared the level of uTWEAK with urine albumin/creatinine ratio in proteinuria detection in patients with LN. Since proteinuria is a component of rSLEDAI scoring system, the study was also included.

^b^Data were presented as the median (interquartile range). Scr: serum creatinine; SLE: systemic lupus erythematosus; LN: lupus nephritis; SLE + LN: SLE patients with active LN; SLE-LN: SLE patients without active LN; rSLEDAI: renal systemic lupus erythematosus disease activity index; uTWEAK: urinary TNF-like weak inducer of apoptosis; sTWEAK: serum TNF-like weak inducer of apoptosis; OPG: osteoprotegerin; MCP-1: monocyte chemoattractant protein-1; IL-8: interleukin-8; NR: not reported.

### Quality assessment and publication bias

**Figure 2.**
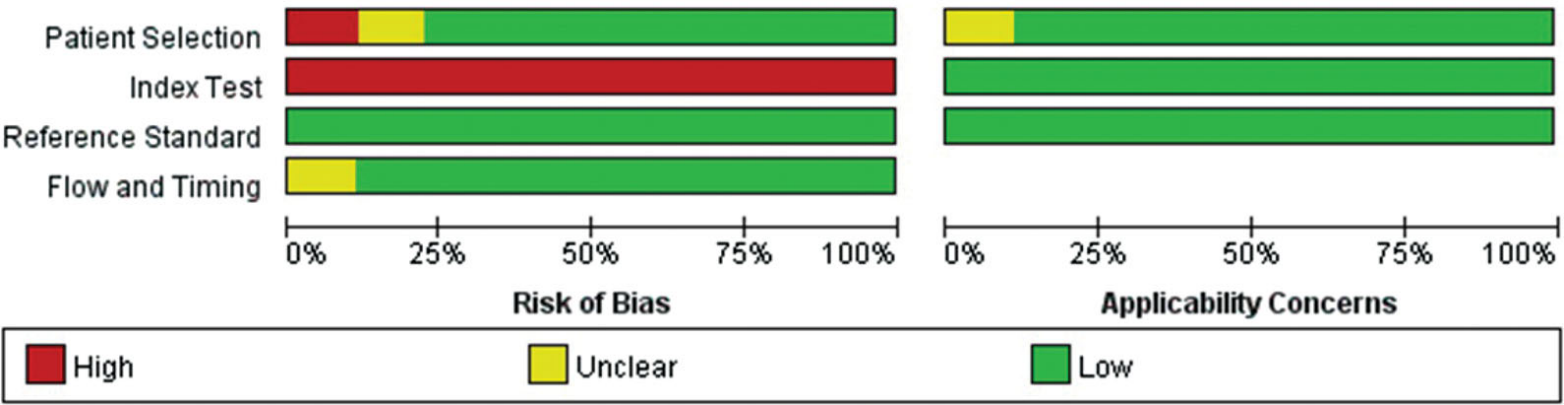
Methodological quality graph.

**Figure 3.**
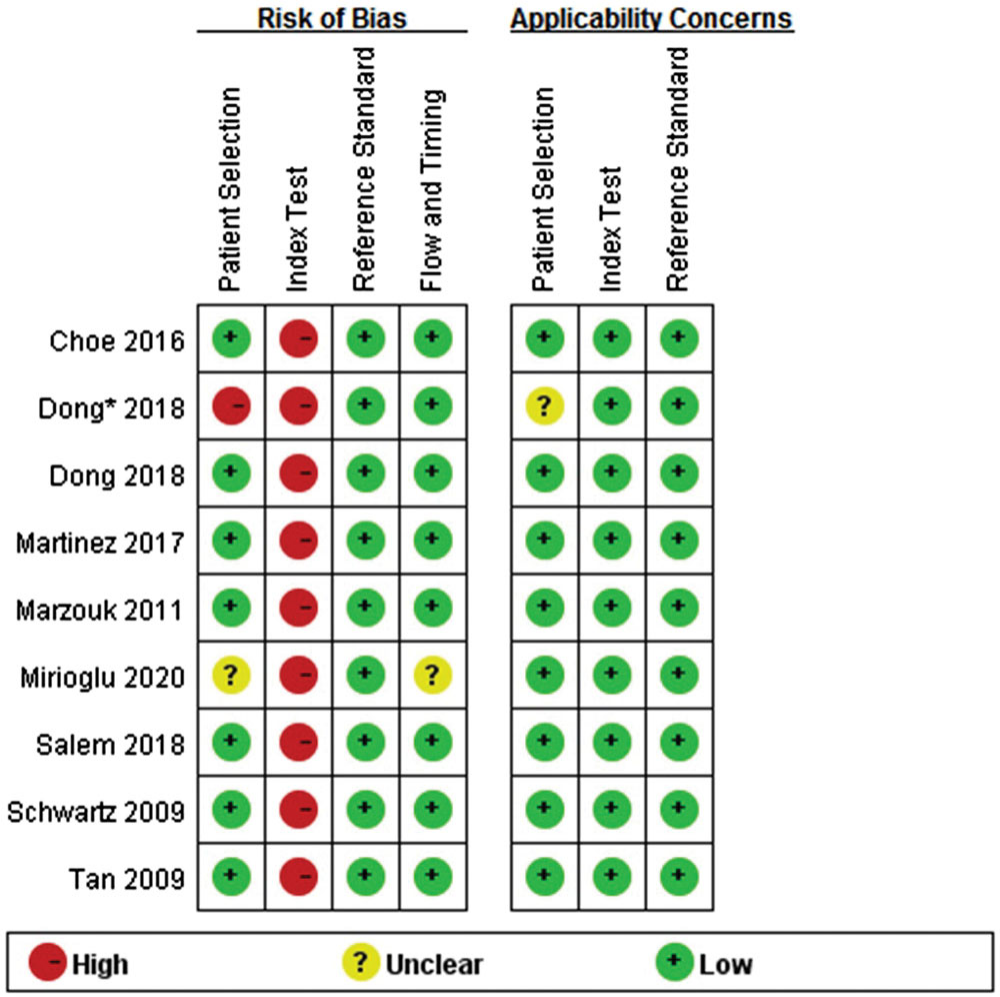
Methodological quality summary. *This study compared the level of uTWEAK with urine albumin/creatinine ratio in proteinuria detection in patients with LN. Since proteinuria is a component of rSLEDAI scoring system, the study was also included. (The asterisk* in the following figures indicates the same.)

**Figure 4.**
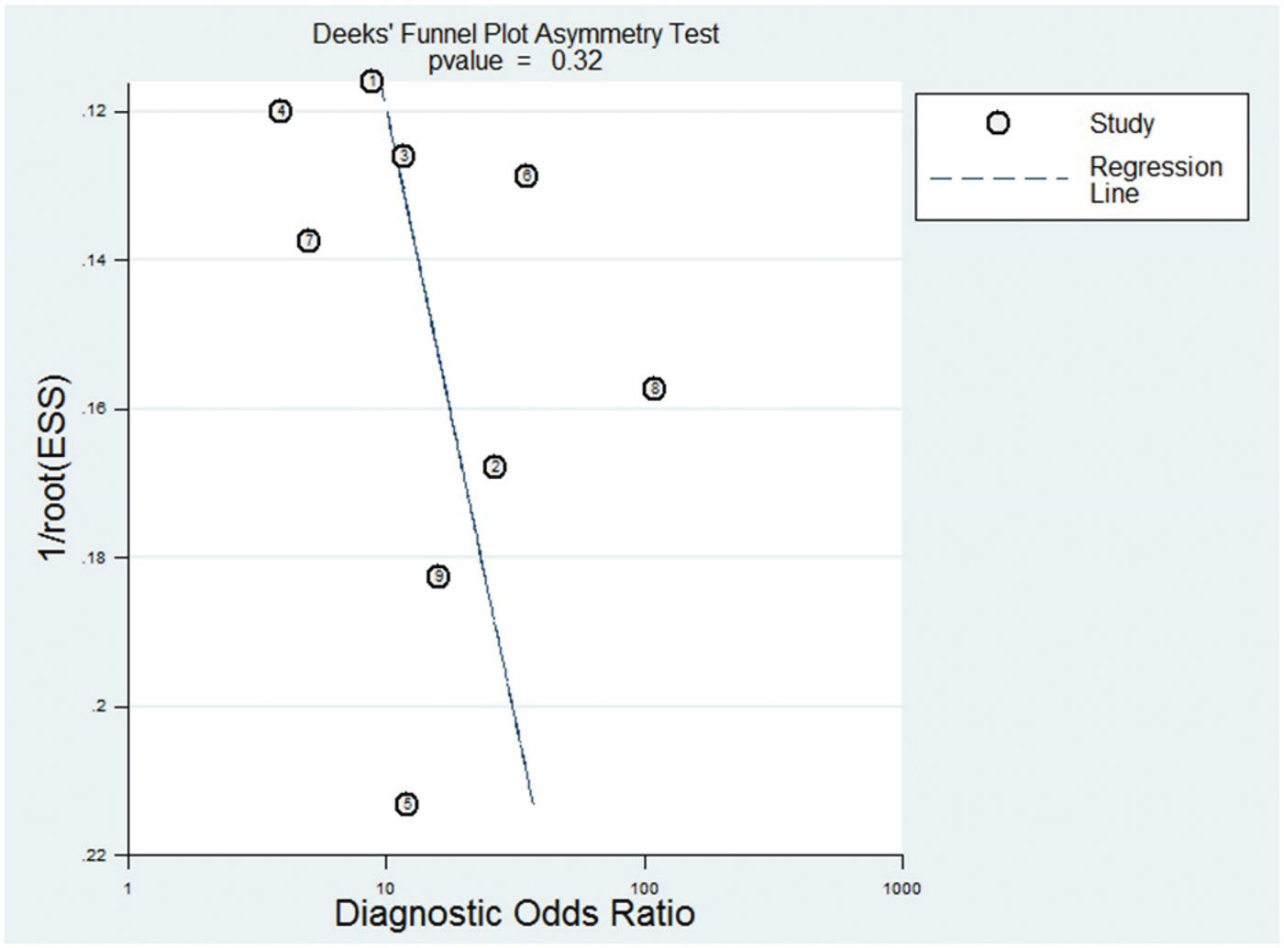
Deeks’funnel plot for detecting publication bias of the included studies.

The results of the QUADAS-2 tool are illustrated in Figures 2, 3. Higher risk was identified in the section of index test, since all studies did not use pre-specified cutoff values but the optimal ones. To assess the potential role of publication bias, the funnel plot method was used. Deeks’ funnel plot revealed no small trial bias of TWEAK in the diagnosis of active LN in the included studies (Figure 4. *p* = .32).

### Data synthesis

Extracted data from the included studies are shown in Table 2, including TP/FP/FN/TN, optimal cutoff value, sensitivity, specificity and AUC. Urine or serum TWEAK was measured by enzyme-linked immunosorbent assay (ELISA) in all studies.

1. Pooled sensitivity

There was a significant heterogeneity among the studies (*p* < .0001, I^2^ = 84.3%), so the random-effects model was used for the meta-analysis. The overall pooled sensitivity of TWEAK for the diagnosis of active LN was 0.69 (95% CI, 0.63–0.75) (Figure 5(A)).

1. Pooled specificity

There was a significant heterogeneity among the studies (*p* < .0001, I^2^ = 77.7%), so the random-effects model was used for the meta-analysis. The overall pooled specificity of TWEAK for the diagnosis of active LN was 0.77 (95% CI, 0.71–0.82) (Figure 5(B)).

1. Pooled positive likelihood ratio (PLR)

There was a significant heterogeneity among the studies (*p* = .003, I^2^ = 66.2%), so the random-effects model was used for the meta-analysis. The overall pooled PLR of TWEAK for the diagnosis of active LN was 3.31 (95% CI, 2.05–5.35) (Figure 5(C)).

1. Pooled negative likelihood ratio (NLR)

There was a significant heterogeneity among the studies (*p* < .0001, I^2^ = 72.2%), so the random-effects model was used for the meta-analysis. The overall pooled NLR of TWEAK for the diagnosis of active LN was 0.38 (95% CI, 0.26–0.55) (Figure 5(D)).

1. Pooled diagnostic odds ratio (DOR)

There was no significant heterogeneity among the studies (*p* = .360, I^2^ = 9.0%), so the fixed-effects model was used for the meta-analysis. The overall pooled DOR of TWEAK for the diagnosis of active LN was 10.89 (95% CI, 6.73–17.63) (Figure 5(E)).

1. Summarized receiver operating characteristic (SROC) curve

**Figure 6.**
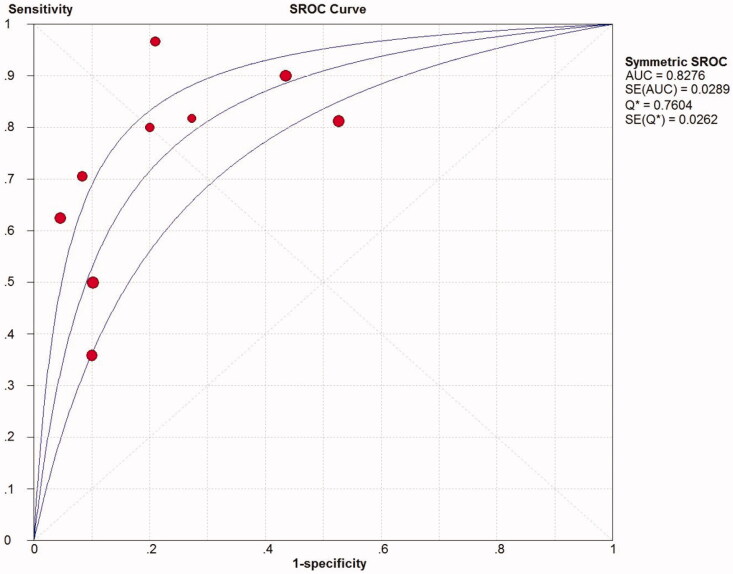
SROC curve for quantitative analysis of TWEAK in the diagnosis of active LN. SROC: summary receiver operating characteristic; AUC, area under the receiver operating characteristic curve; SE, standard error.

The SROC curve was calculated by sensitivity against (1-specificity). Figure 6 depicted an AUC (standard error, SE) of 0.8276 (0.0289) with a Q* value (SE) of 0.7604 (0.0262), indicating a high diagnostic accuracy of TWEAK for predicting active LN.

Figure 5.Diagnostic sensitivity, specificity, positive likelihood ratio (PLR), negative likelihood ratio (NLR) and diagnostic odds ratio (DOR) of TWEAK in predicting active LN across all included studies. CI: confidence interval; df: degree of freedom; LR: likelihood ratio; OR: odds ratio.

**Figure:**
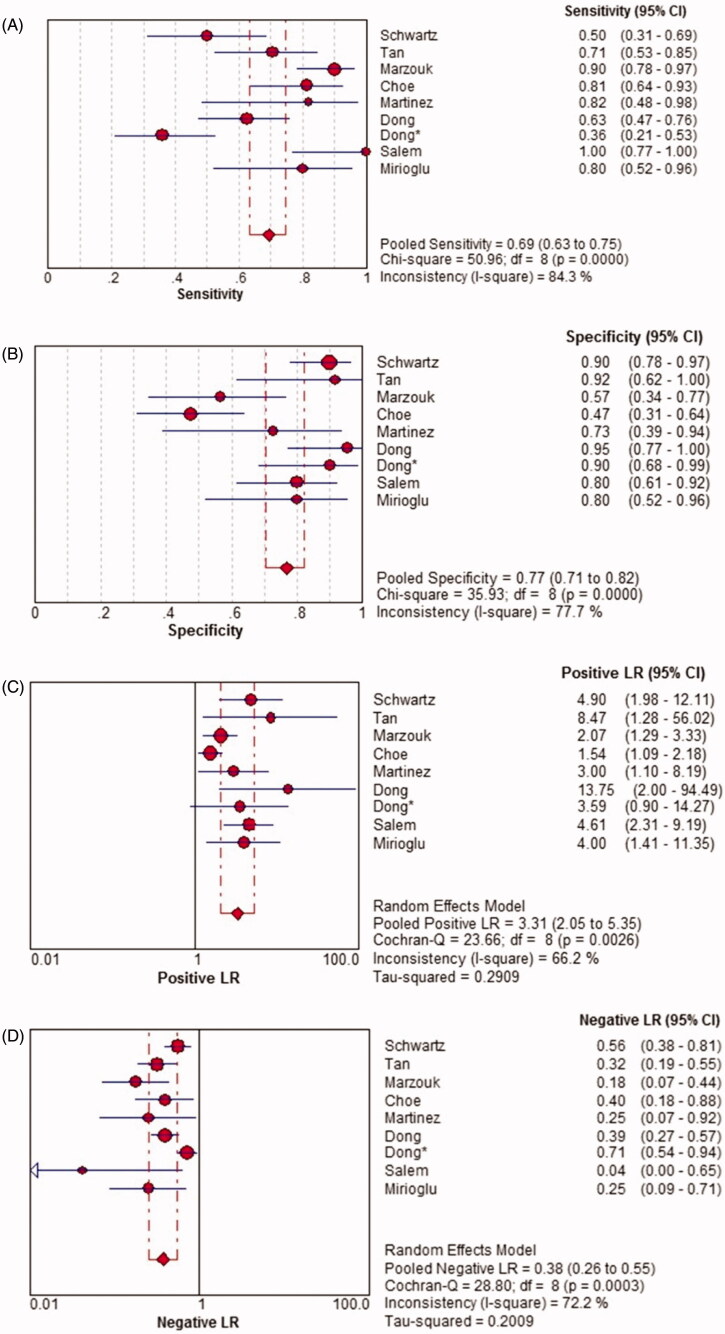

**Figure:**
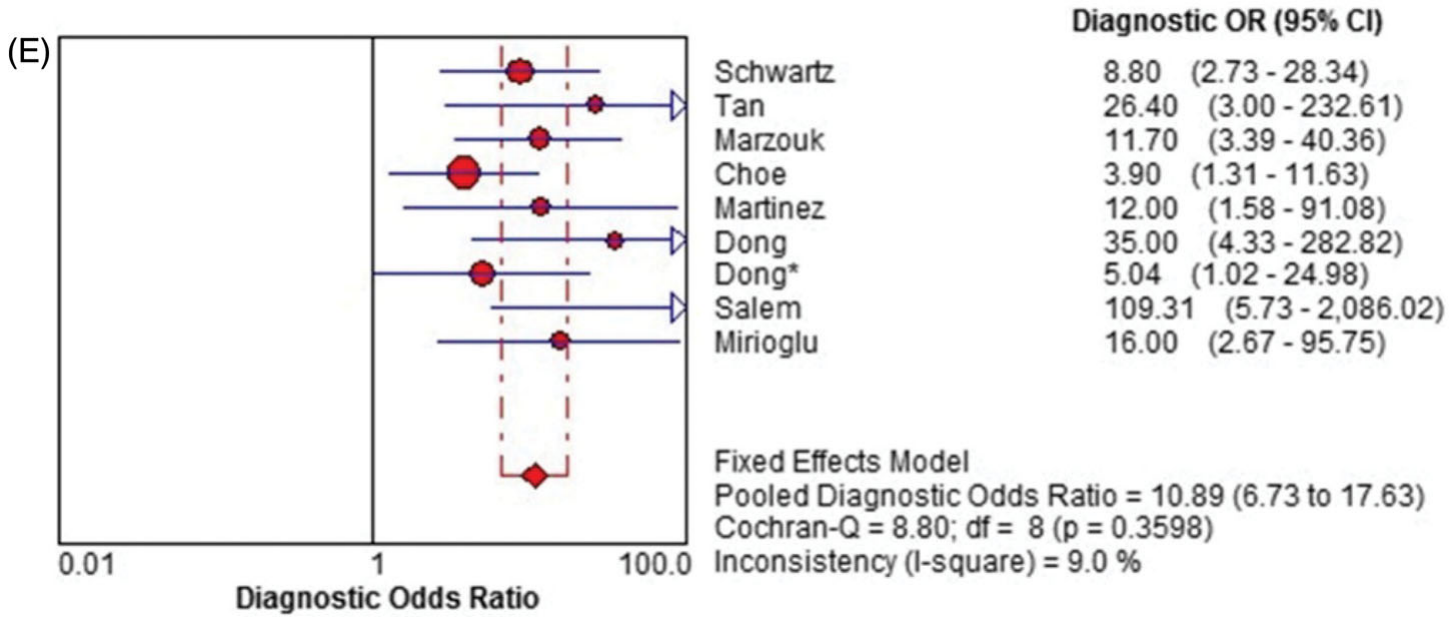

**Table 2.** Diagnostic accuracies of the included studies for TWEAK to predict active LN from lupus patients.

Author, year	Sample type	Measurement method	TP	FN	FP	TN	TWEAK cutoff	Sensitivity	Specificity	AUC (95% CI)
Schwartz, et al. 2009	Urine	ELISA	15	15	5	44	13 pg/mg Cr	50%	90%	0.724 (NR)
Tan, et al. 2009	Urine	ELISA	24	10	1	11	4.34 pg/mg Cr	69.70%	92.31%	0.790 (NR)
El-shehaby, et al. 2011	Urine	ELISA	45	5	10	13	9.1 pg/mg Cr	89%	56%	0.816 (NR)
Choe, et al. 2016^b^	Serum	ELISA	26	6	20	18	395.0 pg/mL	81.3%	47.5%	0.648 (0.52–0.78)
Martínez, et al. 2017	Urine	ELISA	9	2	3	8	4.91 pg/mg Cr	81%	75%	0.876 (0.75–0.99)
Dong, et al. 2018	Urine	ELISA	30	18	1	21	12.53 pg/mg Cr	62.22%	93.33%	0.815 (0.699–0.930)
Dong, et al. 2018^a^	Urine	ELISA	14	25	2	18	26.95 pg/mg Cr	36.7%	88.9%	0.626 (0.427–0.825)
Salem, et al. 2018	Urine	ELISA	14	0	6	24	8.22 pg/mg Cr	100%	80%	0.96 (NR)
Mirioglu, et al. 2020^b^	Serum	ELISA	12	3	3	12	1542.2 ng/mL	80%	80%	0.796 (0.622–0.969)

^a^This study compared the level of uTWEAK with urine albumin/creatinine ratio in proteinuria detection in patients with LN. Since proteinuria is a component of rSLEDAI scoring system, the study was also included.

^b^uTWEAK levels were also measured in the indicated studies. However, there was lack of mandatory indexes regarding uTWEAK in distinguishing patients with active LN in the studies. TWEAK: TNF-like weak inducer of apoptosis; LN: lupus nephritis; TP: true positive; FN: false negative; FP: false positive; TN: true negative; AUC: area under the receiver operating characteristic curve; CI: confidence interval; ELISA: enzyme-linked immunosorbent assay; Cr: creatinine; NR: not reported.

### Subgroup analysis

Next, we performed subgroup analysis of the included studies (Table 3). As shown by the data, there was significant heterogeneity in specificity across all groups. Subgroup analysis revealed that the sensitivity of TWEAK to predict active LN in patients with a rSLEDAI score > 4 was higher compared with that in patients with a score > 0 (0.85 versus 0.66). Moreover, judging from the comparisons of DOR and AUC, the diagnostic value of TWEAK to predict active LN was much higher in the former subgroup (DOR: 19.00; AUC: 0.90 versus DOR: 8.90; AUC: 0.79). The DOR of urinary TWEAK was substantially higher than that of serum TWEAK in predicting active LN (12.40 versus 6.56). Since only two studies included mandatory statistics regarding serum TWEAK, AUC could not be calculated.

**Table 3.** Subgroup analysis of the included studies.

	*N*	Sensitivity (95% CI)	I^2^ (%)	Specificity (95% CI)	I^2^ (%)	PLR (95% CI)	I^2^ (%)	NLR (95% CI)	I^2^ (%)	DOR (95% CI)	I^2^ (%)	AUC
rSLEDAI score												
> 0	4	0.66 (0.57–0.75)	65.1	0.77 (0.68–0.84)	88.8	3.80 (1.29–11.22)	82.8	0.44 (0.35–0.57)	4.1	8.90 (3.79–20.98)	29.6	0.79
> 4	3	0.85 (0.76–0.91)	80.4	0.74 (0.61–0.84)	68.3	3.39 (1.59–7.22)	61.2	0.20 (0.08–0.55)	62.6	19.00 (6.38–56.55)	7.7	0.90
Sample type												
Urine	7	0.67 (0.60–0.73)	87.3	0.83 (0.77–0.89)	63.2	3.71 (2.33–5.91)	38.0	0.38 (0.25–0.60)	77.3	12.40 (6.65–23.13)	0	0.86
Serum	2	0.81 (0.67–0.91)	0	0.57 (0.42–0.70)	79.8	2.21 (0.84–5.84)	69.8	0.33 (0.18–0.63)	0	6.56 (1.73–24.94)	42.6	NA

rSLEDAI: renal systemic lupus erythematosus disease activity index; N: number of studies; CI: confidence interval; PLR: positive likelihood ratio; NLR: negative likelihood ratio; DOR: diagnostic odds ratio; AUC: area under the receiver operating characteristic curve; NA: not available.

### Correlations between TWEAK and various parameters

Table 4 summarizes the results of the correlations between TWEAK and various indicators, including laboratory, pathological and clinical parameters. Seven studies reported the correlations of TWEAK with rSLEDAI. Four studies investigated the associations between TWEAK and renal function (serum creatinine and blood urea nitrogen). A total of six studies included the relevance between TWEAK and proteinuria (either 24 h urine protein or urine protein/creatinine ratio).

**Table 4.** Correlations between TWEAK and laboratory/pathological/clinical parameters in the included studies.

Author, year	Sample type	Scr	BUN	24h UP	UP/Cr	C3	C4	Anti-dsDNA	CRP	ESR	BAI	BCI	rSLEDAI	Hematuria	Pyuria
Schwartz, et al. 2009	Urine	NR	NR	NR	NR	NR	NR	NR	NR	NR	NR	NR	+	NR	NR
Tan, et al. 2009	Urine	NR	NR	NR	NR	NR	NR	NR	NR	NR	+	NS	NR	NR	NR
El-shehaby, et al. 2011	Urine	NR	NR	+	NR	–	–	NR	NR	NR	NR	NR	+	+	+
Choe, et al. 2016	Serum Urine	NS NS	NS NS	NR NR	+ NS	NS NS	NS NS	NS NS	NS NS	+ NS	NR NR	NR NR	+ NS	NR NR	NR NR
Martínez, et al. 2017	Urine	NS	NS	+	NR	–	–	NR	NR	NR	NR	NR	NR	NR	NR
Dong, et al. 2018	Urine	NR	NR	NR	NR	NR	NR	NR	NR	NR	+	NS	+	NR	NR
Dong, et al. 2018^a^	Urine	NS	NS	+	NR	NR	NR	+	NR	NR	NR	NR	+	NR	NR
Salem, et al. 2018	Urine	NS	NS	NS	NR	NS	NS	NS	NS	NS	NR	NR	+	+	+
Mirioglu, et al. 2020	Serum Urine (ng/ml) Urine (ng/mgCr)	NR NR NR	NR NR NR	+ NS NS	NR NR NR	– NS NS	NS NS NS	NR NR NR	NR NR NR	NR NR NR	NR NR NR	NR NR NR	+ + NS	NR NR NR	NR NR NR

^a^This study compared the level of uTWEAK with urine albumin/creatinine ratio in proteinuria detection in patients with LN. Since proteinuria is a component of rSLEDAI scoring system, the study was also included. Scr: serum creatinine; BUN: blood urea nitrogen; 24 h UP: 24 h urine protein; UP/Cr: urine protein/creatinine ratio; C3, C4: serum complement components C3 and C4; Anti-dsDNA: anti-double stranded DNA; CRP: C-reactive protein; ESR: erythrocyte sedimentation rate; BAI: biopsy activity index; BCI: biopsy chronicity index; rSLEDAI: renal systemic lupus erythematosus disease activity index; +: positive correlation; -: negative correlation; NS: no significance; NR: not reported.

## Discussion

As LN is one of the most serious manifestations of SLE leading to significant morbidity and mortality among patients, early recognition of the disease is of vital importance. Conventional laboratory parameters cannot ideally differentiate renal activity from renal damage in LN [37]. Therefore, multiple novel biomarker candidates, such as anti-neutrophil cytoplasmic antibody, serum or urinary cytokines, chemokines, cell adhesion molecules, calcium-binding proteins and microRNAs, have been proposed for detecting early renal flares or disease severity in LN in recent years [38–42]. In spite of the identification of these putative biomarkers that track histopathologic activity, their sensitivity and specificity are unsatisfactory. Another concern is that seldom have these biomarkers been evaluated in a prospective manner to determine if they truly reflect the dynamic changes in the disease course [43]. Moreover, the cost-effectiveness of a biomarker (i.e., lower costs and quicker diagnosis) should be taken into account as some of the proposed candidate markers cannot be measured routinely in the hospital.

As a member of the TNF superfamily, TWEAK and its specific receptor FN14 can regulate a number of biological processes such as cell proliferation, migration, differentiation, tissue regeneration, angiogenesis and induction of inflammatory cytokines [44]. Transient activation of TWEAK/FN14 facilitates physiologic tissue repair and regeneration following acute injury, whereas excessive activation drives pathological tissue responses, leading to inflammation and cell death [45]. TWEAK has been shown to induce NF-κB signaling and participate in immune-mediated inflammatory conditions, such as SLE, rheumatoid arthritis, inflammatory bowel disease and psoriasis [46,47]. TWEAK contributes to kidney inflammation by promoting cytokine production in different renal cells (tubular cells, mesangial cells, podocytes and fibroblasts) through canonical and non-canonical NF-κB activations [48]. Furthermore, TWEAK activation also contributes to renal fibrosis in LN, a final common pathway leading to ESRD [49].

Recently, TWEAK has been proposed as a promising biomarker of active LN in patients with SLE [20,21,30–36]. Schwartz et al. reported that lupus patients with active LN had higher levels of urinary TWEAK (uTWEAK) compared to lupus patients with non-active LN [21]. Mirioglu et al. suggested that uTWEAK level was correlated with rSLEDAI. However, after normalization with urine creatinine values, uTWEAK was not associated with rSLEDAI. Moreover, uTWEAK level was not significantly different between active renal and extra-renal SLE. Further analyses revealed that only serum TWEAK (sTWEAK) was able to distinguish patients with active LN from those without LN [20]. Similarly, Choe et al. concluded that sTWEAK was a potential biomarker for renal involvement in SLE, whereas uTWEAK was not [31]. The levels of TWEAK in patients with different LN classes have also been investigated. Tan and Marzouk et al. noted that the levels of uTWEAK did not vary significantly in patients with different biopsy classification [30,36]. Choe et al. found that there was no statistical difference regarding sTWEAK level between proliferative and membranous nephritis [31]. Studies by Dong et al. revealed that LN patients with class V had higher levels of uTWEAK compared with those with other classes and that the overall difference of average uTWEAK was significant in various pathological groups [33,34]. In an effort to investigate the diagnostic utility of TWEAK, two included studies compared uTWEAK to other routinely used biomarkers of LN. Schwartz et al. found that previously used biomarkers such as anti-dsDNA, C3 and C4 did not have the same discriminatory power as uTWEAK in identifying LN patients from SLE patients [21]. Similarly, Dong et al. suggested that C3 and C4 might not be specific enough to renal disease activity in SLE patients [33].

Current studies focusing on the diagnostic performance of TWEAK were inconsistent because of single-center design or small sample size. In this context, we therefore conducted the present study. To our knowledge, this might be a novel meta-analysis which assessed the diagnostic value of TWEAK for active LN. Our findings suggested that TWEAK was elevated in patients with LN and might serve as a promising predictor, with DOR of 10.89 (95% CI, 6.73–17.63) and AUC (SE) of 0.8276 (0.0289). The pooled PLR and NLR of the included studies were 3.31 (95% CI, 2.05–5.35) and 0.38 (95% CI, 0.26–0.55), indicating a satisfactory diagnostic performance. There was no publication bias of the included studies (*p* = .32). Further subgroup analysis revealed that TWEAK had a higher diagnostic value to predict active LN in the subgroup of patients with a rSLEDAI score of more than 4. Urinary TWEAK seemed to be a more promising biomarker as compared to serum TWEAK based on its higher DOR.

However, there were some limitations in this meta-analysis. First, the number of the included studies was limited and most of the studies had a small sample size. Second, a majority of the enrolled studies used rSLEDAI score in the diagnosis of active LN; however, due to the unreliable nature of urinalysis, this could have constituted an inherent limitation of this study. For instance, false positive proteinuria might be encountered in the setting of SLE, which is not necessarily indicative of renal disease [50]. Besides, hematuria might be a clinically irrelevant finding, especially in the male population [51]. Therefore, urinalysis results should be interpreted prudently in patients with LN. After all, renal biopsy is still the gold standard for delineating activity and chronicity indices [52]. Third, because of the multiple mechanisms contributing to LN, it was unlikely that one single biomarker could be sufficient to predict renal disease activity in SLE patients. Furthermore, the applied cutoff values varied among different studies, which might be attributable to differences in study protocols (e.g., different sample types, patient eligibility criteria, definition of active LN, etc). The tendency of studies to use the optimal thresholds might have overestimated the diagnostic accuracy of TWEAK. According to the book *Biostatistics and Epidemiology: A Primer for Health Professionals*, the choice of cutoff point is influenced by many factors [53].

In conclusion, the present meta-analysis provided evidence that TWEAK might be a potential biomarker for patients with active LN. Future cross-sectional and longitudinal studies are needed to confirm its diagnostic value, as well as to establish more definite cutoff for active LN in the clinical practice.
